# Comparative Chromosome Painting and NOR Distribution Suggest a Complex Hybrid Origin of Triploid *Lepidodactylus lugubris* (Gekkonidae)

**DOI:** 10.1371/journal.pone.0132380

**Published:** 2015-07-06

**Authors:** Vladimir A. Trifonov, Alessio Paoletti, Vincenzo Caputo Barucchi, Tatiana Kalinina, Patricia C. M. O’Brien, Malcolm A. Ferguson-Smith, Massimo Giovannotti

**Affiliations:** 1 Institute of Molecular and Cellular Biology SB RAS, Novosibirsk, Russia; 2 Novosibirsk State University, Novosibirsk, Russia; 3 Dipartimento di Scienze della Vita e dell’Ambiente, Università Politecnica delle Marche, Ancona, Italy; 4 Cambridge Resource Centre for Comparative Genomics, University of Cambridge, Cambridge, United Kingdom; 5 Consiglio Nazionale delle Ricerche, Istituto di Scienze Marine Sezione Pesca Marittima,Ancona, Italy; University of Florence, ITALY

## Abstract

Parthenogenesis, unisexuality and triploidy are interesting but poorly studied phenomena occurring in some reptile species. The mourning gecko (*Lepidodactylus lugubris)* represents a complex of diploid and triploid parthenogenetic mostly all-female populations (males occur quite rarely) widely distributed in coastal areas of the Indian and Pacific Oceans. Here, we study karyotypes of a male and two female *L*. *lugubris* (LLU) triploid individuals (3n = 66) using comparative painting with *Gekko japonicus*, *Hemidactylus turcicus* and *H*. *platyurus* chromosome specific probes to visualize the homologous regions and to reveal genus specific rearrangements. Also, we applied a 28S ribosomal DNA probe and Ag-staining to detect nucleolus organizer regions (NORs). Our results suggest that the karyotype of *L*. *lugubris* underwent a chromosome fission and a fusion after its divergence from a common ancestor of the *Gekko-Hemidactylus* group. The NORs were found to be located on one out of three homologs on each of LLU8, LLU15 and LLU18, thus further confirming a hybrid origin of triploid individuals. It seems that three different bisexual populations might have contributed to the origin of this triploid parthenogenetic population. We postulate that the heterozygosity in NOR localization is maintained in the triploid clone studied by the absence of recombination as described in whiptail lizards. The pattern of NOR localizations and homologous regions in males and females, as well as the absence of other detectable karyotypic differences, suggest that males arise spontaneously in all female populations and do not arise from independent hybridizations with different species.

## Introduction

Although unisexuality is found in approximately 80 taxa of vertebrates (fish, amphibians and reptiles), and new unisexual species are still being described, reproduction in the complete absence of males is restricted to reptiles and is referred to as true parthenogenesis [1], in which an oocyte develops in the absence of spermatozoa [2]. This form of parthenogenesis (also known as “obligate parthenogenesis”) is naturally occurring and has been found in more than 40 species of lizards (Agamidae, Chamaeleonidae, Gekkonidae, Gymnophthalmidae, Teiidae, Scincidae, Lacertidae, Xantusiidae) and snakes (Typhlopidae), and is intimately associated with a preceding interspecific hybridization between related bisexual species ([2], and references therein). Facultative parthenogenesis, which was thought until recently to occur only in captive animals (birds, reptiles and sharks), has been described in two wild snake species, supporting the hypothesis that non-hybrid parthenogenesis is more common in squamates than previously thought [3].

Obligate parthenogenesis shows its highest variety in the family Gekkonidae, with seven all females species belonging to five different genera [2]. Among these lizards, the mourning gecko, also known as the common smooth-scaled gecko (*Lepidodactylus lugubris)*, has long been a target for studies focusing on parthenogenesis, karyosystematics and evolution of sex determination mechanisms in vertebrates. Since the first description of parthenogenesis in this species [4] it has become a model species for cytogenetic and physiological studies. The mourning gecko is the most widespread parthenogenetic species, occurring in Southeast Asia, Australia, most islands of the Pacific, and Central America [2], and its natural populations are either diploid (2N = 44) or triploid (3N = 66). These latter were suggested to be derived from a backcrossing of diploid *L*. *lugubris* with males of the parental species [5].

Volobouev and Pasteur [6] have detected chromosomal polymorphism in some diploid female populations and even suggested that the largest pair represented sex chromosomes. Later the same group proposed a hybrid origin of diploid parthenogenetic populations based on a high heterogeneity of homologous chromosomes within individuals [7]. Radtkey et al [8] identified *L*. *moestus* as a maternal ancestor of the species, and a still undescribed species, *Lepidodactylus sp*., which is distributed from French Polynesia to the Marshall Islands, as a paternal ancestor. Besides, they demonstrated an ongoing generation of different *L*. *lugubris* clones and hypothesized that all previously reported sexual populations of *L*. *lugubris* were misidentified. In some cases these phenotypic males were sterile hybrids between *L*. *lugubris* and the ancestral sexual species [8][9][10]. In fact, populations of parthenogenetic squamates are usually all-female, and hybrid males in the F1 generation have not been described [2]. However, males are reported to emerge occasionally from all-female populations, both natural and captive [11][12][13]. Study of spermatogenesis in the rare mourning gecko males has demonstrated that the mechanisms necessary for parthenogenesis in females are detrimental to spermatogenesis and lead to infertility in males [2].

Here we have used several different molecular cytogenetic techniques to characterize general and sex specific karyotypic features of *L*. *lugubris*: i) comparative chromosome painting was used to detect the homologous syntenic segments between *L*. *lugubris* (LLU) and some other geckos (*Gekko japonicus* (GJA), *Hemidactylus turcicus* (HTU) and *H*. *platyurus* (HPL)) and to reconstruct chromosome evolution among the studied geckos; ii) C-banding and Comparative Genomic Hybridization (CGH) were applied to detect differences between the genomes of males and females and to identify possible differences between heteromorphic sex chromosomes; and iii) FISH with a ribosomal probe and AgNOR staining were performed to study the karyotypic distribution of nucleolus organizer regions and their possible role as indicators of the species origin.

## Materials and Methods

### Ethical Statement

The tail tips were obtained by two owners of animal collections using induced caudal autotomy, which is a natural anti-predatory phenomenon, and none of the animals was sacrificed. The authors of this article did not play any role in manipulations with animals. Induced caudal autotomy as a naturally occurring phenomenon usually does not lead to severe wounds or severe bleeding, nevertheless the owners of the animals used disinfectant treatments of geckos' tail stumps to prevent infection. The owners of animals gave the authors tissue samples (not live animals) and provided their consent for the experimentation. It was not necessary to get an approval of the Institutional Animal Care and Use Committee (IACUC) or any relevant ethics body since this study on animal tissues complies with article 2e of D.L. 26/2014 of the Italian Government derived from Directive 2010/63/EU of the European Parliament and of the Council (article 1, paragraph 5e) on the protection of animals used for scientific purposes.

### Species Sampled

Captive bred *L*. *lugubris* were used for this study. The four males were reported to be born from parthenogenetic, captive bred females, and were unlikely to be related to each other, as opposed to the two females who shared the same parent. The fact that the female parents of the males studied here were born in captivity greatly lessens the chances of them having been in contact with males of other species. Sex of the studied individuals was determined by visual inspection with a 10x magnification lens of external anatomical features such as preanal and femoral pores and hemipenal bulges, which are present in males and absent in females. The samples were kindly provided by two owners of personal collections.

### Metaphase Preparations

Metaphase preparations of four male and two female *L*. *lugubris* were made from primary fibroblast cultures. The tissue from tale tips was minced and grown in RPMI1640 (Gibco, USA), supplemented with 20% fetal bovine serum (Gibco), 2 mM L-Glutamine (Gibco), penicillin (100 units/ml) and streptomycin (100 μg/ml) (Gibco). Cultures were incubated at 30°C in an incubator in the presence of 5% CO_2_. Chromosome preparations were made following standard procedures that included a 6 hours incubation with KaryoMAX Colcemid (Gibco) (75 ng/ml) followed by 30 min hypotonic treatment in 0.075 M KCl at 30°C, and fixation in 3:1 methanol/glacial acetic acid.

### Karyotyping

Metaphases were stained with 4’,6-diamidino-2-phenylindole (DAPI; Vector Laboratories), images captured with a Leica Leitz DMRBE fluorescence microscope equipped with JAI CV-M4+CL monochrome camera. Chromosomes of all individuals studied were arranged according to their length from the largest to the smallest. Karyotypes were reconstructed using the software Leica CytoVision version 7.2 (Leica Microsystems).

### Chromosome-Specific Probes

Preparation and labeling of *Gekko japonicus*, *Hemidactylus turcicus*, and *H*. *platyurus* chromosome-specific painting probes are described previously [14].

### Cross-Species Chromosome Painting

Sixteen chromosome specific probes of *G*. *japonicus* and 14 probes of *H*. *platyurus* as well as some probes of *H*. *turcicus* were hybridized onto chromosomes of a male (further referred to as “male A”) and two female individuals (further referred to as “female A” and “female B”) of *L*. *lugubris*. Images were captured and processed using the Video TesT system (St Petersburg, Russia) and a ProgResMF CCD camera mounted on an Olympus BX-60 microscope.

### Localization of Major Ribosomal DNA Clusters

A pBR322-derived plasmid containing a 3kb-genomic fragment of human 28S RNA genes (from the collection of the Department of Biodiversity and Genome Evolution IMCB SB RAS) was labeled by nick translation kit (Invitrogen) with either biotin-16-dUTP (Roche) or digoxigenin-11-dUTP (Roche) and detected using avidin-FITC (Life Technologies) or anti-digoxigenin-rhodamine (Roche), respectively. Dual-color FISH with chromosome specific probes was used to determine the NOR-bearing chromosomes.

### AgNOR and C-Banding

C-banding and AgNOR-staining were performed according to previously published protocols [15][16]. Images were captured and processed using the software CytoVision version 7.2 (Leica Microsystems, Gateshead, UK) and JAI CV-M4+CL 1.4 megapixel progressive scan monochrome camera (JAI, Glostrup Copenhagen, DK) mounted on a Leica Leitz DMRBE (Leica Microsystems) fluorescence microscope.

### Comparative Genomic Hybridization (CGH)

DNA was isolated from fibroblast cultures of the *L*. *lugubris* male A and the female A using DNeasy Blood & Tissue Kit (Qiagen). For each male and female 10 ng of DNA was amplified and labeled using the WGA1 kit (Sigma); the male DNA library was labeled with biotin-16-dUTP (Roche) and the female DNA library was labelled with digoxigenin-11-dUTP (Roche). Fifty ng of each probe was ethanol precipitated and reconstituted in 12 μl of hybridization buffer. CGH was performed as described previously [17].

## Results

### Karyotypes

**Fig 1:**
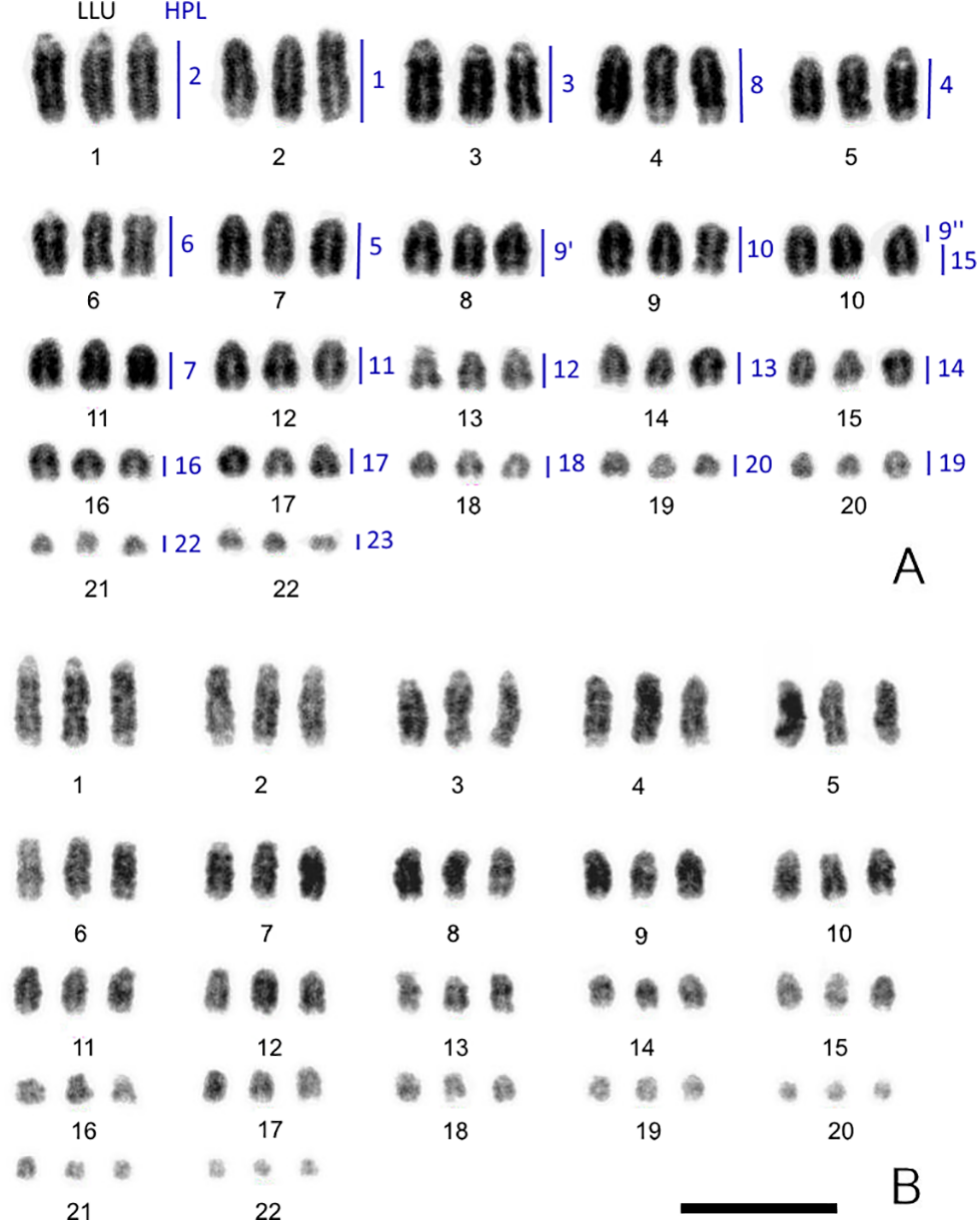
Inverted DAPI-stained karyotypes of the mourning gecko (*Lepidodactylus lugubris*, LLU). 3n = 66. (A) male A and (B) female A. Homologies between LLU and *Hemidactylus platyurus* (HPL) revealed by chromosome painting are indicated on male karyotype. Scale bar, 10 μm.

The karyotypes of the six animals studied here each contained 66 chromosomes (Fig 1), a chromosomal number that represents the triploid condition for this species (2N = 44 in diploid clones). This was confirmed by chromosome painting experiments (see below). Chromosomes were arranged in karyotypes from largest to smallest, as was suggested by Volobouev and Pasteur [6]. No obvious heteromorphism in either males or females was detected.

### Chromosome Painting

**Fig 2:**
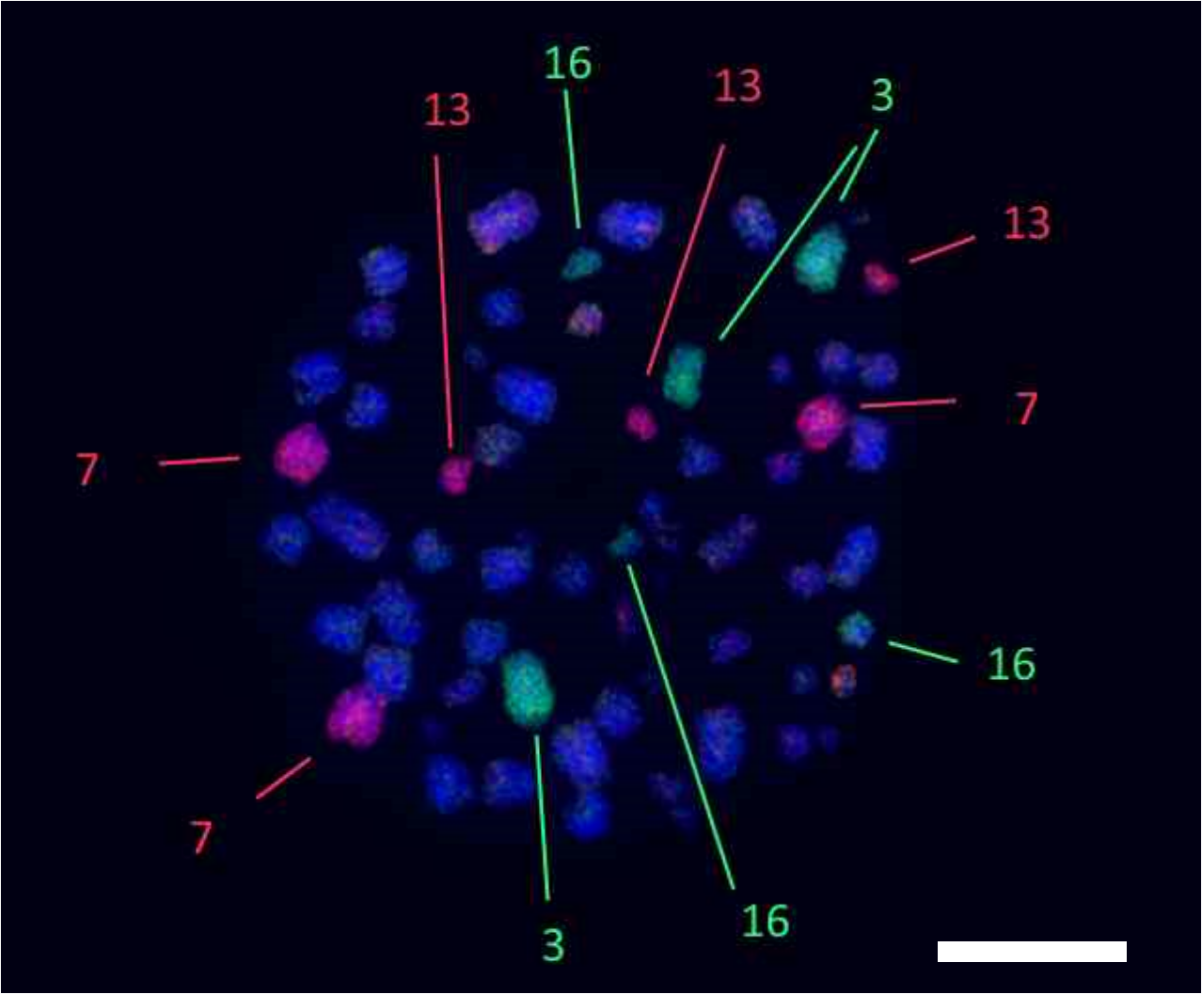
Example of *Gekko japonicus* painting probes localization onto chromosomes of *Lepidodactylus lugubris*. Painting probes specific to chromosomes GJA1 (green) and GJA2 (red) each paints two triplets of LLU (female B) chromosomes: GJA1—LLU3 and LLU16; GJA2 – LLU7 and LLU13. Scale bar, 10 μm.

We hybridized the full sets of chromosome specific probes of *G*. *japonicus* and *H*. *platyurus* as well as some *H*. *turcicus* probes (HTU16, HTU19+20, and HTU21 were used to resolve some ambiguous results) onto chromosomes of *L*. *lugubris* male A, female A and female B. As some of the paint probes contained more than one chromosome, the identification of specific chromosome homologies in some cases required painting with probes from one or other of the three species so that those chromosomes within the probe that were not homologous could be eliminated. Most chromosomes were found to be conserved *in toto* between *L*. *lugubris*, *H*. *platyurus*, *G*. *japonicas*, and *H*. *turcicus* (resulting in three homologs per metaphase painted by the same probe). The probe containing chromosome HPL15 painted the distal part of LLU10 (the same results were obtained using the probe GJA12) (S1 Fig). The probe containing a mixture of chromosomes HPL7, 8, 9, 10 painted four LLU triplets completely (LLU4, 8, 9, 11) and additionally the proximal part of LLU10. The probe containing a mixture of chromosomes GJA7, 8, 9 painted LLU5, 8, 11 and additionally the proximal part of LL10 (S1 Fig). To discriminate a chromosome that underwent fission in LLU, we applied a probe containing chromosomes GJA7 and 8 and found signals only on LLU5 and 11. Thus we concluded that the chromosome homologous to GJA9 (HPL9) underwent fission in LLU. We did not reveal any homology for HPL21 (HTU19). The probes specific to chromosomes GJA1 and GJA2 painted two triplets each in LLU (similar results were obtained in HPL [14]). The probe containing GJA chromosomes 4 and 5 painted three triplets in LLU (LLU1, 6, 20). Results of painting are presented in Table 1, Fig 1A, Fig 2 and S1 Fig.

**Table 1:** Homologous regions between several gekkonid species. Homology between chromosomes of *Lepidodactylus lugubris* (LLU), *Gekko japonicus (GJA)*, *Hemidactylus turcicus* (HTU), and *H*. *platyurus* (HPL). Homologies between GJA, HTU and HPL have been identified previously [14].

LLU chromosome	GJA chromosome	HTU chromosome	HPL chromosome
1	4	6	2
2	3	2	1
3	1q	1	3
4	6	3	8
5	8	4	4
6	5dist	8	6
7	2q	5	5
8	9part	9part	9part
9	10	10	10
10prox	9part	9part	9part
10dist	12	15	15
11	7	7	7
12	11	11	11
13	2p	12	12
14	13	13	13
15	14	14	14
16	1p	16	16
17	16	18	17
18	15	17	18
19	17	20	20
20	5p-qprox	1prox	19
21	18	21	22
22	19	22	23
?	?	19	21

In three samples subjected to comparative paining experiments we detected length polymorphism in nine homologous triplets (in triplets of LLU4, 5, 6, 7, 8, 12, 13, 14, 16 two homologs usually were equal in size and the third homolog was smaller).

### FISH with 28S Probe and AgNOR Banding

**Fig 3:**
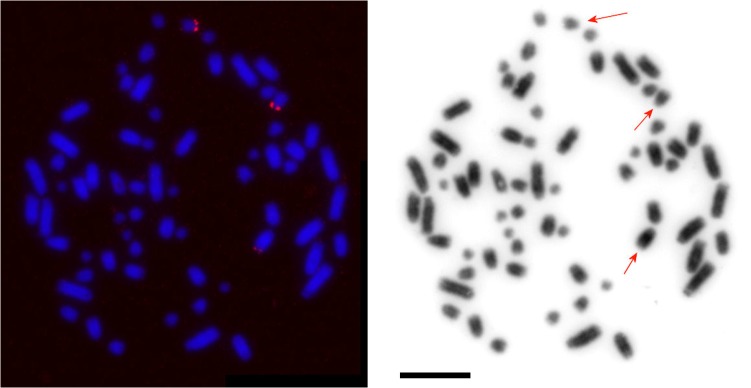
Localization of ribosomal DNA clusters in the mourning gecko (*Lepidodactylus lugubris*). A partial metaphase of the LLU male A painted with human rDNA probe. Arrows indicate NORs in p-arms of two small but different in size chromosomes and in the distal part of the q-arm of a large chromosome. Scale bar, 10 μm.

**Fig 4:**
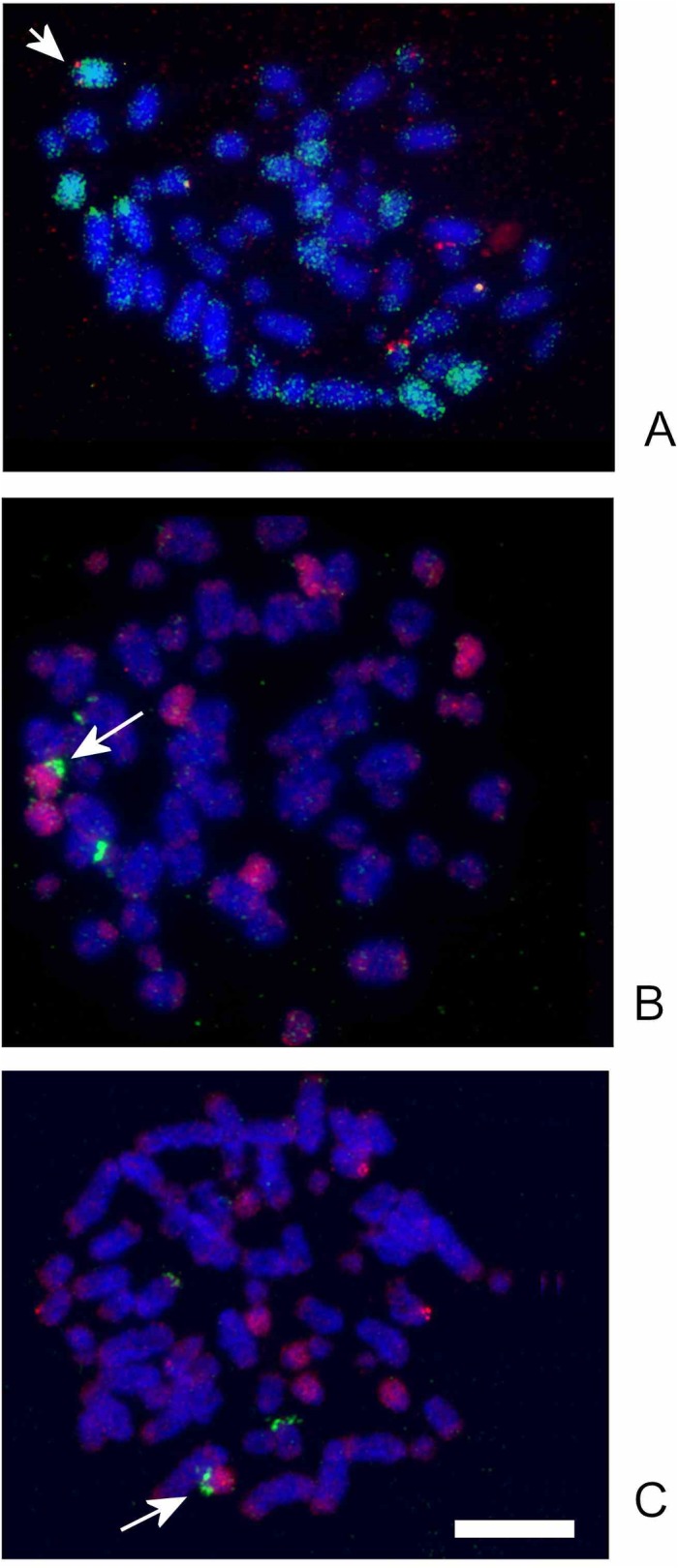
Mapping of ribosomal RNA clusters on *Lepidodactylus lugubris*. (A) Co-localization of the probe containing chromosomes GJA7+8+9 (green, paints LLU5, 8, 11, 10prox) and ribosomal probe (red) on LLU8 in female A (indicated by an arrow). (B) Co-localization of a probe containing GJA13 and GJA14 (red, paints LLU14 and LLU15) and ribosomal probe (green) on LLU15 (indicated by an arrow) in female B. (C) Co-localization of a probe containing a mixture of GJA15+16 (red, paints LLU17 and LLU18) and ribosomal probe (green) on LLU18 (indicated by an arrow) in male A. Scale bar, 10 μm.

We applied FISH with the human 28S ribosomal probe to detect ribosomal DNA clusters in male A, female A and female B. Our results showed the presence of three blocks in the karyotypes of all animals studied: two larger blocks were localized in p-arms of two small (LLU13-LLU19) acrocentric chromosomes and a smaller signal was detected in the subtelomeric region of a medium size chromosome (LLU6-LLU10). It is noteworthy that all three ribosomal cluster bearing chromosomes differed in size, assuming that only one of three homologs retained the ribosomal block in each case (Fig 3). We applied dual colour FISH with differently labeled GJA or HPL probes and a ribosomal probe to determine the NOR-bearing chromosomes precisely. We found that the largest NOR-bearing chromosome was one of the three LLU8 homologs, while LLU15 and LLU18 (only one homolog of each) carried the block in p-arms (Fig 4 and S2 Fig).

**Fig 5:**
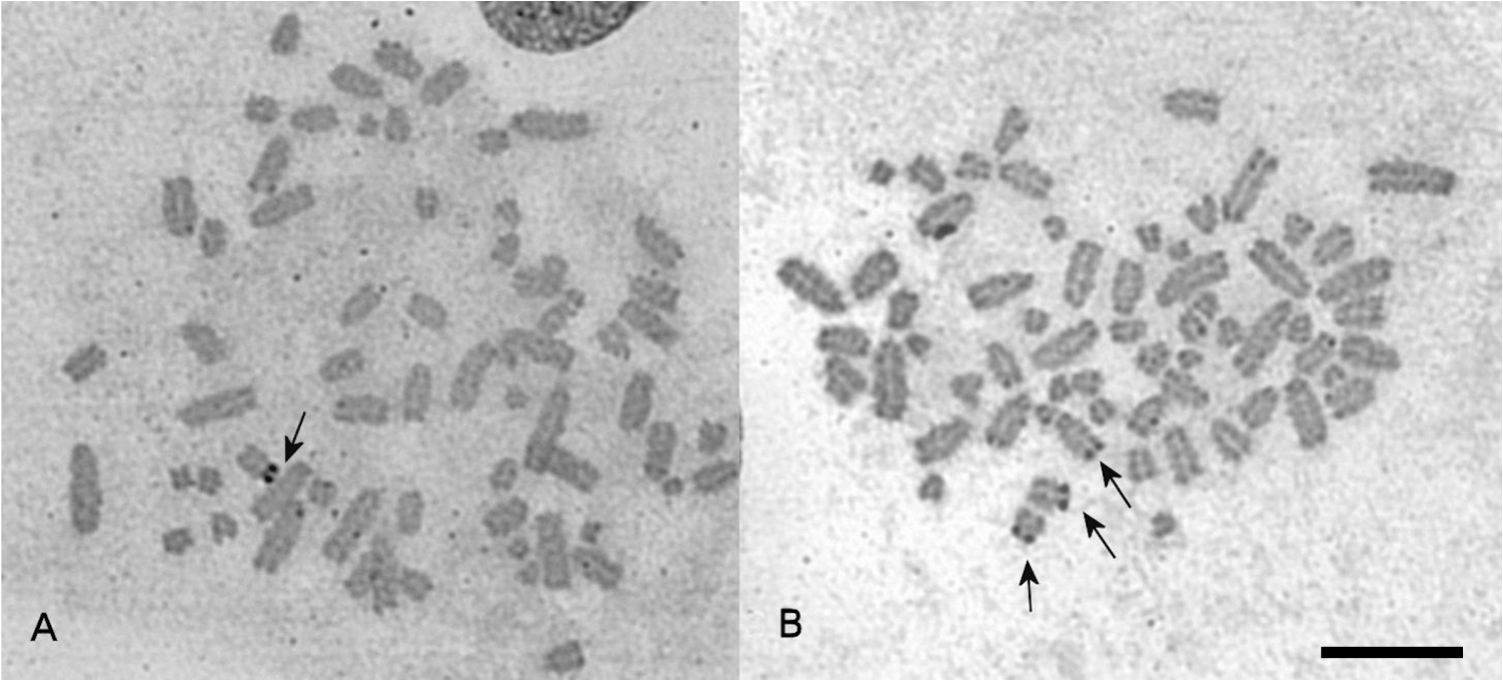
AgNOR staining of *Lepidodactylus lugubris* chromosomes. (A) LLU female B and (B) LLU male D. There is a polymorphism in NOR activity revealed by Ag-staining: only a single chromosome carries an active NOR in female B, but all three NORs are revealed in male D. Scale bar, 10 μm.

AgNOR staining revealed some variability in the number of active NOR sites. In particular, a single active NOR on LLU15p was detected in females A, B and in the male A, whereas three active NORs were detected in the other three males (B, C and D) on LLU8q, LLU15p and LLU18p (Fig 5).

### C-Banding

**Fig 6:**
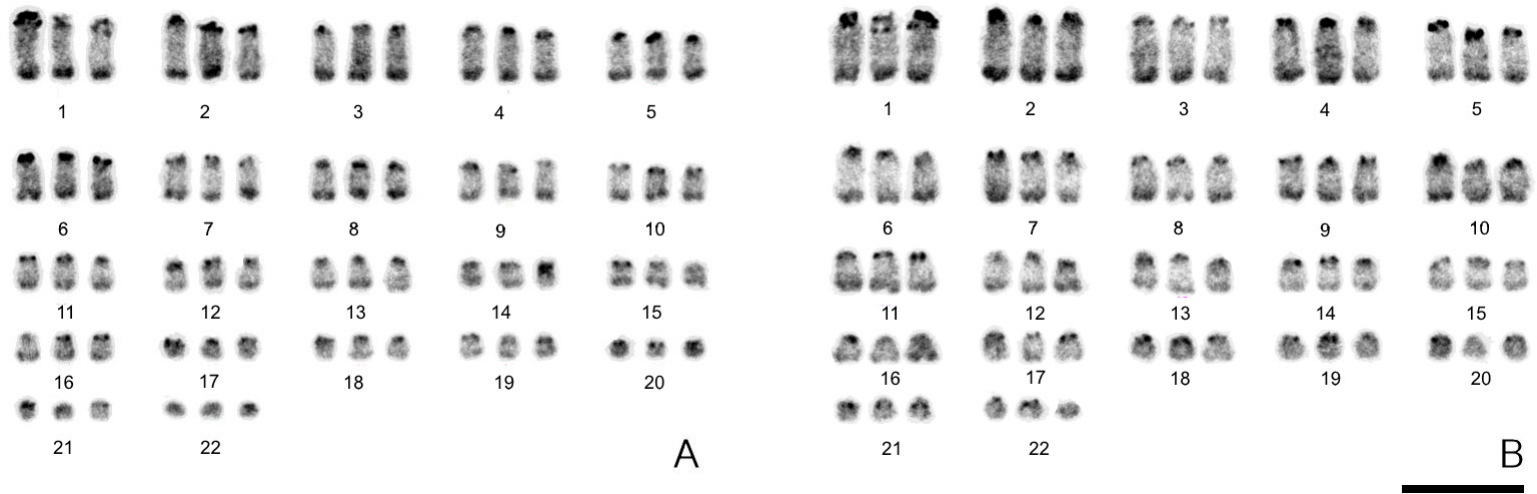
C-banding of *Lepidodactylus lugubris* chromosomes. (A) female B and (B) male A. Pericentromeric and subtelomeric heterochromatic regions are dark-staining. Scale bar, 10 μm.

Heterochromatic material is clearly visible at pericentromeric regions on all chromosomes and at subtelomeric regions of the first 16 triplets of chromosomes. No heteromorphism was detected (Fig 6).

### Comparative Genomic Hybridization

We simultaneously hybridized labeled male (biotin) and female (digoxigenin) LLU genomic DNA onto chromosomes of LLU male and female and found no differently stained regions in any of the animals studied (results not shown).

## Discussion

### Comparative Chromosome Painting of *Lepidodactylus lugubris*. Karyotype Evolution within the *Gekko-Lepidodactylus-Hemidactylus* Group

Comparative chromosome painting has demonstrated that most chromosomes of *L*. *lugubris* were hybridised by single chromosomes of *H*. *platyurus* (2n = 46). We have found only a single fission (HPL9 synteny is split into LLU8 and LLU10prox segments) and a single tandem fusion (presence of blocks HPL9part and HPL15 on LLU10). Homology with HPL21 was not detected (previously we did not find it in two other geckos—*G*. *japonicus* and *H*. *frenatus* (HFR), either) [14].

**Fig 7:**
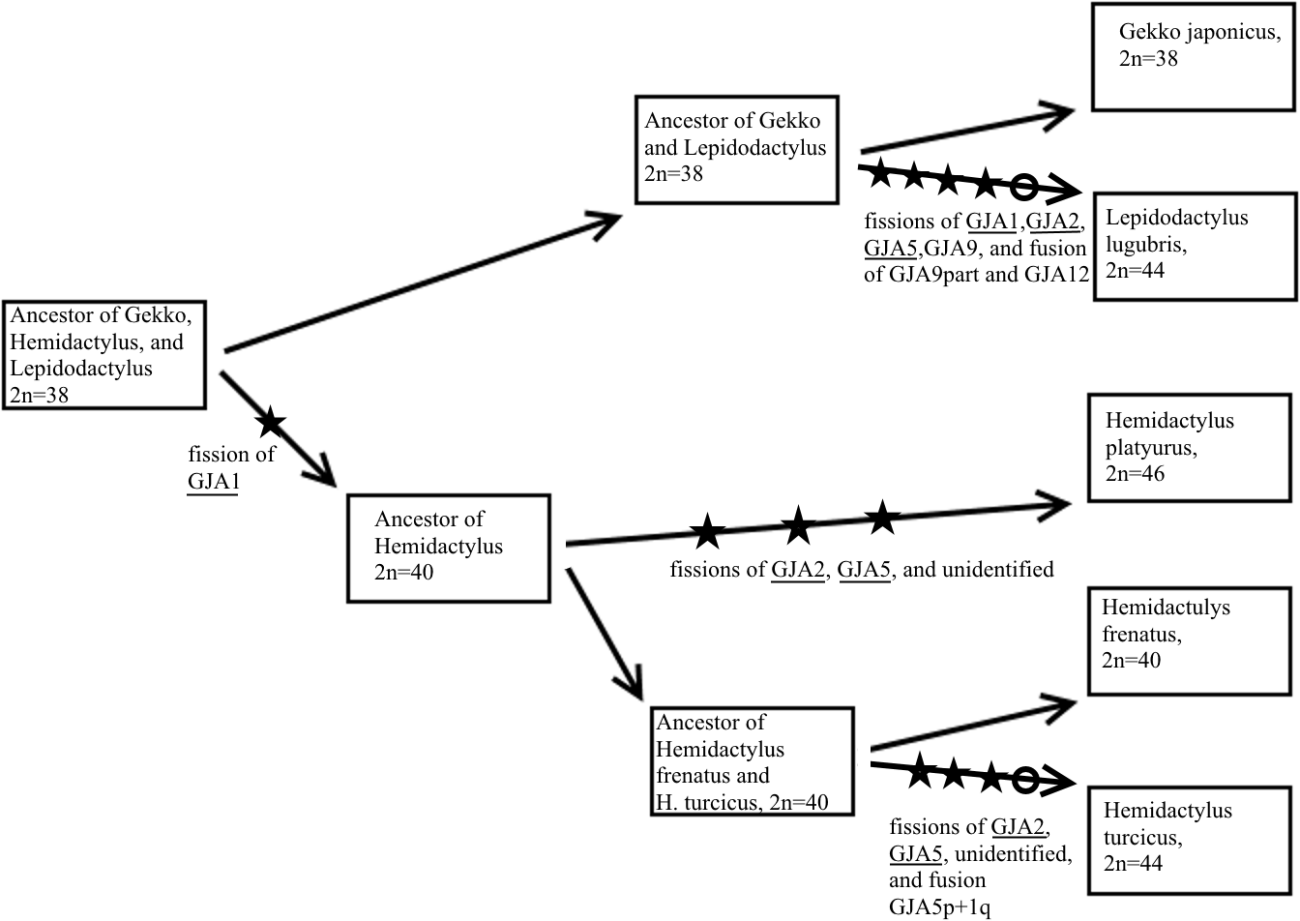
A scenario of karyotype evolution within *Gekko-Lepidodactylus- Hemidactylus* group. Stars indicate chromosome fissions and circles indicate chromosome fusions. The recurrent chromosome rearrangements are underlined. The tree is based on previously published data [19].

According to molecular phylogenies *Lepidodactylus* is more closely related to the genus *Gekko* [18] [19] than to *Hemidactylus*. However, we observed similar karyotypic features of *L*. *lugubris* and *H*. *platyurus* on the one hand, as well as two common fusions (GJA2p+GJA2q = HFR1p+HFR1q and GJA5pqprox+GJA5qdist = HFR2p+HFR2q) both in the *Gekko* lineage and in *H*. *frenatus*. Previously we suggested that the syntenies GJA2 = HFR1 and GJA5 = HFR2 were ancestral [14], and a recent paper of Pokorna et al [20] demonstrates the conservation of GJA2 and GJA1 syntenies across different families of Gekkota. This means that our suggestion about the ancestral state of the *Gekko* karyotype is still valid and high karyotype similarities between *L*. *lugubris* and *H*. *platyurus* on the one hand and between *Gekko* and *H*. *frenatus* on the other hand result from homoplasies. These homoplasies include frequent breakpoint reuse on ancestral elements homologous to GJA1, 2 and GJA5. Fissions of these chromosomes occurred independently in three gekkonid lineages–LLU, HPL and HTU (Fig 7).

### Localization of NORs and the Origin of Triploid Populations

Different species of geckos seem to have quite a high variation in the distribution of nuclear organizer regions. Since chromosome painting has not yet become routine in gekkonid cytogenetics, NORs have only been precisely located in a few species. In our previous paper we reported localization of NORs in p-arms of *H*. *platyurus* chromosome 2 [14], which is homologous to LLU1. Schmid and Guttenbach [21] have localized NORs in the telomeric region of *Gekko gecko* GGE4 (also homologous to LLU1). Shibaike et al [22] studied *G*. *hokouensis* and *G*. *japonicas* and detected NORs in GHO19q and GJA17p (karyotypes were arranged according to a nomenclature based on chromosome morphology [23]). According to our classification based on chromosome size [14] NORs in *G*. *japonicus* are located in p-arms of GJA15 (homologous to LLU18), thus one of *L*. *lugubris* NORs has a similar localization in GJA. We localized NORs in the secondary constriction region of chromosome 1q of *G*. *badenii* (previously we used the synonym *“G*. *ulikovskii”* [14]) and *G*. *vittatus* (homologous to LLU3) (S3 Fig). Generally we may conclude that among different species of *Gekko* and *Hemidactylus* genera NORs are usually located on a single chromosome pair with a species specific pattern.

Volobouev et al [7] using AgNOR staining have demonstrated the presence of nucleolar genes in the telomeric regions of q-arms of four different chromosomes (two large sized, one medium sized, one small sized) in a diploid parthenogenetic clone of *L*. *lugubris* and in a telomeric region of the q-arm of the pair 9 in a sexual population (most probably, *L*. *moestus*). Here we found a single homolog bearing ribosomal clusters in the telomeric region of LLU8. LLU8 and LLU9 are similar in size and considering the absence of painting in previous experiments they might have been confused. Thus we may suggest that the NOR bearing LLU8 is derived from *L*. *moestus*, while the p-arm NOR bearing LLU15 and LLU18 might be from two other genomes.

The origin of triploid populations of *L*. *lugubris* was explained by successive hybridization of diploid *L*. *moestus* females and unknown species males, followed by hybridization of F1 females with males of either *L*. *moestus* or unknown species as proposed previously [8].

Here we first show that all three parental genomes differ in NOR localization. This suggests that apart from *L*. *moestus* at least two unknown species might have been involved in the origin of the triploid clone. On the other hand, the three genomes might have come from different populations of the same species (*i*.*e*. *L*. *moestus*) each characterized by a different NOR polymorphism. The polymorphism in size of some autosomes might indicate that two of the parent species (with homologs of similar size) are closely related to each other, but we cannot exclude the possibility that the size difference results from polymorphic heterochromatic regions. We think it is unlikely that the current distribution of ribosomal RNA clusters resulted from translocations of NORs from a single ancestral chromosome triplet onto other chromosomes. The origin of triploid clones from three parental genomes has previously been suggested in several parthenogenetic species from the *Hemidactylus garnotii-vietnamensi* complex [24][25][26]. Our data further corroborate this hypothesis and demonstrate that similar processes might be characteristic for different parthenogenetic lizards. The results of microsatellite analysis have previously demonstrated the presence of three different alleles in several loci and a uniformity of microsatellite patterns among representative of the same clones [27]. Our results propose a role of NORs as candidate markers of parental *Lepidodactylus* species and further research of other clones described in [5] is needed to justify this. Stability of NOR distribution on particular homologs between specimens suggests an absence of recombination between homologs in the once formed triploid clone. We hypothesize that *Lepidodactylus* might have developed a system of premeiotic chromosome doubling followed by pairing and recombination of sister chromosomes to maintain heterozygosity as in the genus *Aspidoscelis* [28]. This mechanism is quite different from the automictic parthenogenesis based on the fusion of egg and polar body, which is accompanied by a reduction of heretozygosity (described in the Comodo dragon [29], sharks [30] [31] and snakes [3] [32]).

The identity of male and female karyotypes at the molecular cytogenetic level (similar C-banding and the absence of visible differences in comparative genomic hybridization experiments (results not shown)) are in agreement with the hypothesis that males arise spontaneously from all female populations of the mourning gecko [2][12][13], and not from separate hybridization events.

## Supporting Information

S1 Fig Localization of *Gekko japonicus* chromosome specific probes on *Lepidodactylus lugubris*. (A) GJA7+8+9 (green, paints LLU5, 8, 11, 10prox) and GJA12 (red, paints LLU10dist) probes onto chromosomes of female B. (B) GJA7+8+9 (green) and GJA4+5 (red, paints LLU1, 6, 20) probes onto chromosomes of female B. (C) GJA11 (green, paints LLU12) and GJA7+8 (red, paints LLU5 and LLU11) probes onto chromosomes of female B. Scale bar, 10 μm.
(TIF)

S2 Fig Mapping of ribosomal RNA clusters on *Lepidodactylus lugubris*. (A) Localization of GJA2 probe (red, paints chromosomes LLU7 and LLU13) and ribosomal probe (green) in female B (NOR-bearing homologs of LLU8, 15 and 18 are indicated by arrows). (B) Co-localization of GJA7+8+9 (red, paints LLU5, 8, 11, 10prox) and ribosomal probe (green) on LLU8 (NOR-bearing homologs of LLU8, 15 and 18 are indicated by arrows) in female B. (C) Localization of GJA11 (green, paints LLU 12) and ribosomal probe (red) indicated by an arrow) in male A. (D) Co-localization of GJA15+16 (red, paints LLU 17 and 18) and ribosomal probe (green) on LLU18 (NOR-bearing homologs are indicated by arrows) in female B. Scale bar, 10 μm.
(TIF)

S3 Fig Localization of ribosomal DNA clusters in the golden gecko (*Gekko badenii*) and in the lined gecko (*G*. *vittatus*). Arrows indicate NORs in the q-arms of the largest chromosome pair. Scale bar, 10 μm.
(TIF)
