# Supplementary figures and images for: Comparative Chromosome Painting and NOR Distribution Suggest a Complex Hybrid Origin of Triploid Lepidodactylus lugubris (Gekkonidae)
Source: PLoS One. 2015 Jul 6;10(7):e0132380. doi: 10.1371/journal.pone.0132380 (PMC4492922; doi:10.1371/journal.pone.0132380)

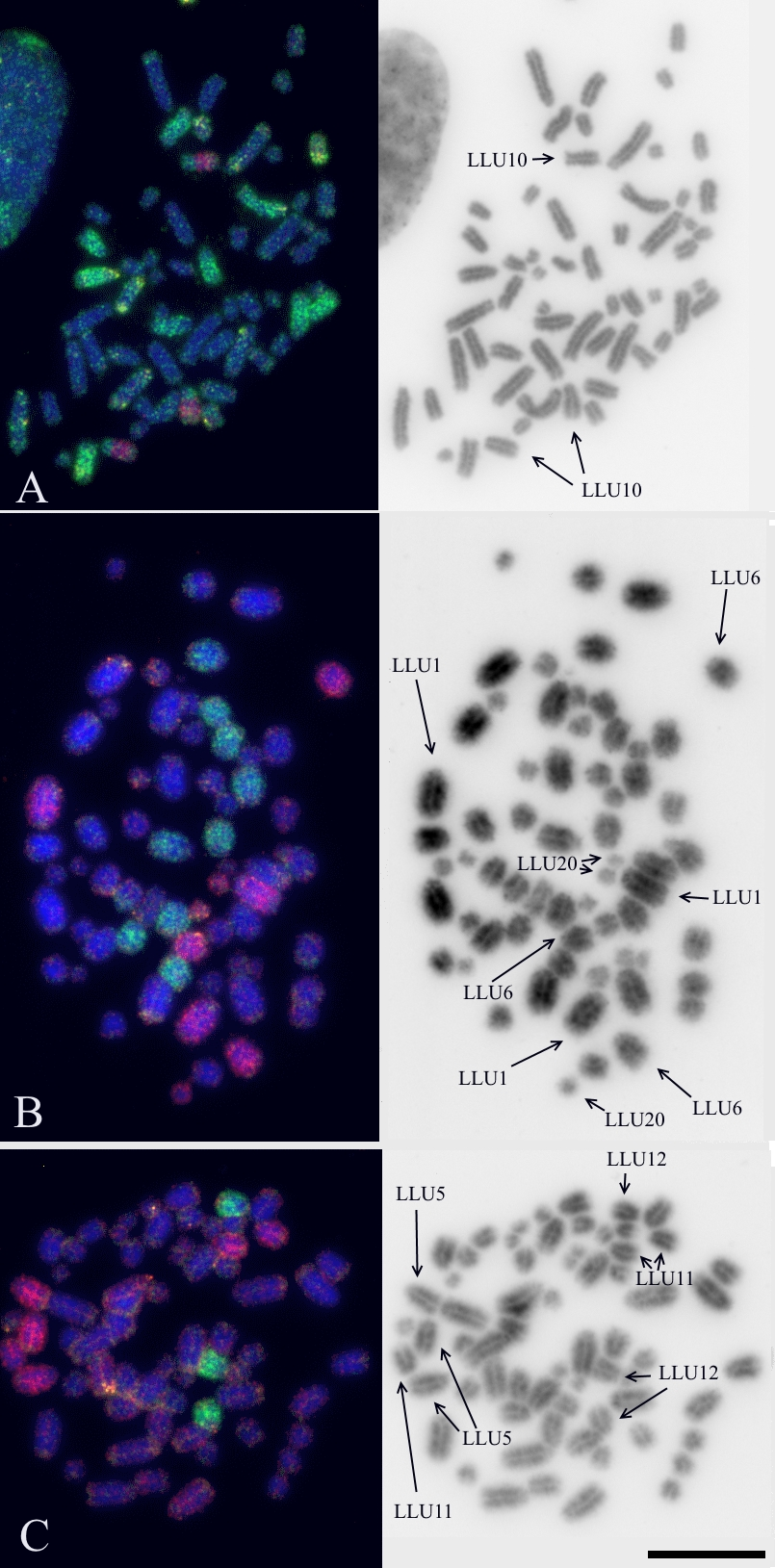

Supplement: S1 Fig — (A) GJA7+8+9 (green, paints LLU5, 8, 11, 10prox) and GJA12 (red, paints LLU10dist) probes onto chromosomes of female B. (B) GJA7+8+9 (green) and GJA4+5 (red, paints LLU1, 6, 20) probes onto chromosomes of female B. (C) GJA11 (green, paints LLU12) and GJA7+8 (red, paints LLU5 and LLU11) probes onto chromosomes of female B. Scale bar, 10 μm. (TIF) [file pone.0132380.s001.tif]

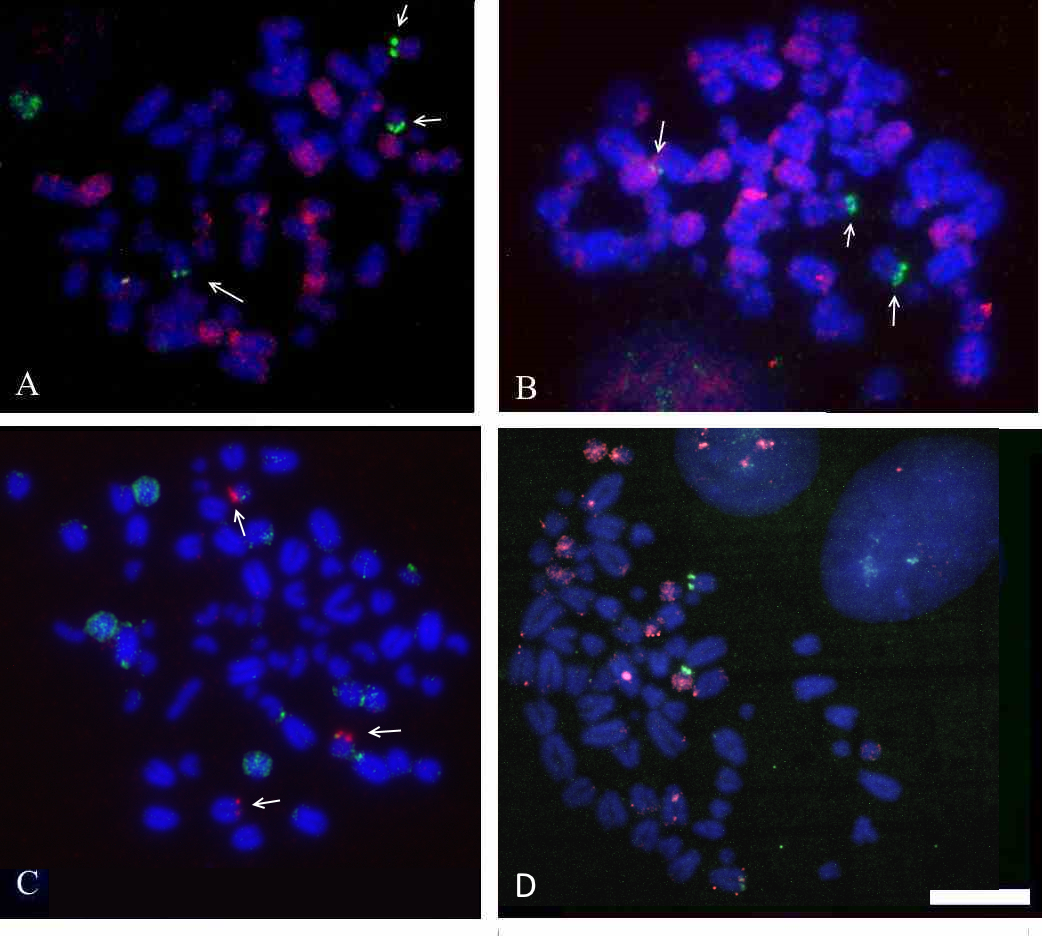

Supplement: S2 Fig — (A) Localization of GJA2 probe (red, paints chromosomes LLU7 and LLU13) and ribosomal probe (green) in female B (NOR-bearing homologs of LLU8, 15 and 18 are indicated by arrows). (B) Co-localization of GJA7+8+9 (red, paints LLU5, 8, 11, 10prox) and ribosomal probe (green) on LLU8 (NOR-bearing homologs of LLU8, 15 and 18 are indicated by arrows) in female B. (C) Localization of GJA11 (green, paints LLU 12) and ribosomal probe (red) indicated by an arrow) in male A. (D) Co-localization of GJA15+16 (red, paints LLU 17 and 18) and ribosomal probe (green) on LLU18 (NOR-bearing homologs are indicated by arrows) in female B. Scale bar, 10 μm. (TIF) [file pone.0132380.s002.tif]

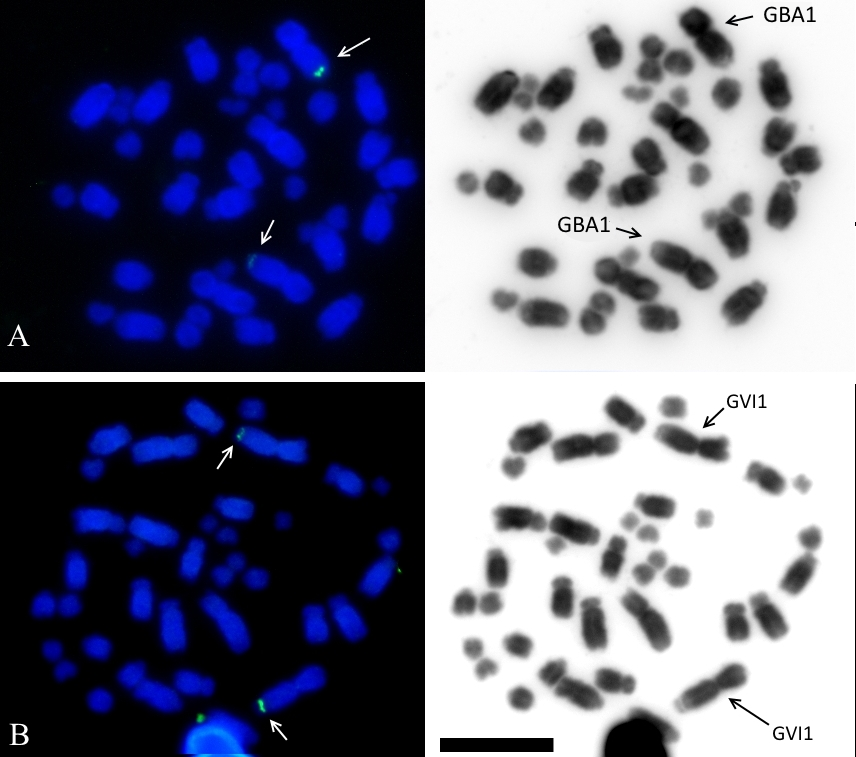

Supplement: S3 Fig — Arrows indicate NORs in the q-arms of the largest chromosome pair. Scale bar, 10 μm. (TIF) [file pone.0132380.s003.tif]
